# Crosstalk and the evolvability of intracellular communication

**DOI:** 10.1038/ncomms16009

**Published:** 2017-07-10

**Authors:** Michael A. Rowland, Joseph M. Greenbaum, Eric J. Deeds

**Affiliations:** 1 Environmental Laboratory, US Army Engineer Research and Development Center, 3909 Halls Ferry Road, Vicksburg, Mississippi 39180, USA; 2 Oak Ridge Institute for Science and Education, Oak Ridge, Tennessee 37830, USA; 3 Center for Computational Biology, University of Kansas, 2030 Becker Dr Lawrence, Kansas 66047, USA; 4 Department of Molecular Biosciences, University of Kansas, 1200 Sunnyside Avenue, Lawrence, Kansas 66047, USA; 5 Santa Fe Institute, 1399 Hyde Park Rd., Santa Fe, New Mexico 87501, USA

## Abstract

Metazoan signalling networks are complex, with extensive crosstalk between pathways. It is unclear what pressures drove the evolution of this architecture. We explore the hypothesis that crosstalk allows different cell types, each expressing a specific subset of signalling proteins, to activate different outputs when faced with the same inputs, responding differently to the same environment. We find that the pressure to generate diversity leads to the evolution of networks with extensive crosstalk. Using available data, we find that human tissues exhibit higher levels of diversity between cell types than networks with random expression patterns or networks with no crosstalk. We also find that crosstalk and differential expression can influence drug activity: no protein has the same impact on two tissues when inhibited. In addition to providing a possible explanation for the evolution of crosstalk, our work indicates that consideration of cellular context will likely be crucial for targeting signalling networks.

## Abstract (web summary)

The evolutionary rationale behind the extensive crosstalk between Metazoan signalling pathways remains elusive. Here the authors provide evidence that crosstalk in the human signalling network evolves as a means to allow efficient diversification of cellular responses to the same signals between different cell types.

Signalling networks allow cells to process information from their environment and respond in appropriate ways to input signals. These networks are generally constructed from a set of interacting proteins; changes in the activity of these proteins across the network transmits the signal from the cell membrane to downstream elements, ultimately resulting (on average) in a particular phenotypic response (for example, proliferation, apoptosis or differentiation). Traditionally, these networks have been organized into ‘canonical pathways’ corresponding to sets of proteins that are involved in the transmission of a specific signal^1^,^2^,^3^,^4^,^5^,^6^,^7^,^8^. For example, the human signalling network includes pathways that are activated by insulin-like growth factor-I (IGF-I), Wnt or apoptotic signals^5^,^6^,^7^,^8^. Although they are often studied separately, these pathways can demonstrate a high degree of ‘crosstalk,’ where proteins that are shared between two pathways cause one pathway’s activity to be modulated by the activity of another^1^,^2^,^9^,^10^,^11^,^12^.

The degree of crosstalk present in signalling networks varies widely across evolution. For instance, bacterial two-component signalling (TCS) networks possess little crosstalk, with most histidine kinases (HKs) acting on a single target (Fig. 1a)^13^,^14^,^15^. We recently demonstrated that this lack of crosstalk is likely a result of the fact that the histidine kinases that make up these networks are generally bifunctional, acting as both kinase and phosphatase for their substrates^16^,^17^,^18^. In contrast, metazoan networks display incredible levels of crosstalk (Fig. 1b): Kirouac *et al*. recently showed that there are more connections between canonical pathways than within them^1^,^2^,^4^,^9^,^10^,^11^,^12^. Crosstalk in metazoan networks is thus so extreme that the individual pathways generally can no longer be discerned once they are combined into a single network (Fig. 1b).

While the kinases and phosphatases typical of metazoan signalling clearly do not share the same enzymatic constraints as bacterial TCS systems^12^,^13^,^14^,^15^,^16^, the fact that they can display crosstalk does not explain why it is so extensive. In this work, we explore the hypothesis that the crosstalk present in metazoan networks has evolved, at least in part, due to the constraints multicellularity has placed on intracellular communication. In particular, metazoans have multiple different cell types, many of which need to react differently to the same stimulus (Fig. 1c). For example, during wound healing endothelial cells construct new blood vessels, fibroblasts establish the new extracellular matrix, and epithelial cells proliferate and migrate to close the skin^19^,^20^,^21^. If metazoan cells contained TCS-like networks (that is, networks with essentially no crosstalk, Fig. 1a), they would need to evolve a new signalling molecule (for example, a new cytokine) and cognate receptor for each combination of cell type/response they needed to control separately. In organisms as complex as mammals, this approach would likely require thousands of unique cytokines, each binding specifically to one of thousands of unique receptors. A TCS-like architecture would also present a major barrier to the evolution of new cell types, since each new cell type would require the evolution of a unique complement of signals and receptors for any novel map between environment and phenotypic response that it needed to exhibit (that is, a set of unique ‘signalling channels’).

We posited that the extensive crosstalk present in metazoan networks, combined with differences in the expression of various nodes in the network in different cell types, might allow those cell types to all respond differently to precisely the same set of signals. This would enable metazoans to encode a wide diversity of responses using a relatively small number of cytokines and receptors. To test this hypothesis, we first created a simple Boolean network with only two inputs and two outputs, and evolved these networks to maximize the number of unique input/output maps the network could exhibit. We found that the evolved networks, which exhibited significant crosstalk and complexity, could generate hundreds of unique input/output maps with only two inputs and two outputs.

To test this idea in a more realistic biological system, we generated a large-scale Boolean network model of the signalling network inside human cells by combining 29 signalling pathways curated in the Kyoto Encyclopedia of Genes and Genomes (KEGG) database^3^. Using available data on protein expression^22^, we found that the architecture of the signalling network changes in different tissues. In the complete signalling network, every input is able to signal to each of the transcription regulating outputs. However, when nodes are removed based upon expression data in various tissues, none of the inputs are able to directly signal to every output. Despite this, the cell-specific subnetworks retain a high degree of input-output interconnectivity when compared to subnetworks in which nodes are expressed at random, indicating that the expression of the signalling proteins in different tissues have likely evolved to maintain extensive crosstalk. As a result of this connectivity, the cell-specific networks demonstrate a much greater diversity in responses to stimuli than either cells with random protein expression or cells with TCS-like networks that have no crosstalk. Interestingly, we found that the tissue-specific networks generally respond quite differently to the inhibition of individual proteins. These results imply that the complex interplay between network topology and gene expression that allows different cell types to respond differently to precisely the same signals has important consequences for the development of drugs that target signalling processes^23^,^24^,^25^.

## Results

### Crosstalk and expression provide a diversity of responses

To demonstrate how network complexity can regulate the cellular decision-making process, we developed an evolvable Boolean network model that begins with a simple network consisting of two inputs that each activates a single, cognate output (Supplementary Methods). At each evolutionary step, the model can perform one of three possible modifications: (1) add an edge, (2) flip the sign of an edge, or (3) add an intermediate node (Fig. 2a). New networks are evaluated according to the number of unique input–output maps they can generate, as described below; if that number increases, the modified network is ‘kept’ by the algorithm and used for the next set of modifications. If that number does not increase, the new network is discarded and the algorithm attempts another random modification of the previous topology.

**Figure 1:**
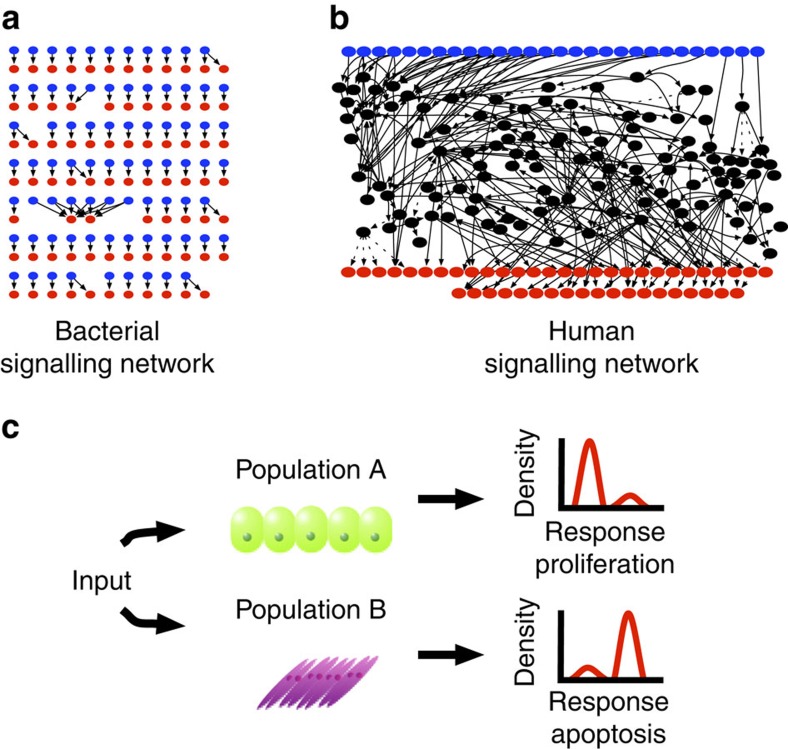
Bacterial TCS versus human signalling networks. (**a**) Diagram of the bacterial TCS network. Blue nodes represent the inputs, sensor HKs, and red nodes represent the outputs, Response Regulators (RRs). Note the highly isolated nature of the TCS network: the majority of inputs only point to one, or at most two RR’s. (**b**) Diagram of the human signalling network. This representation draws from six of the 29 canonical pathways from the KEGG pathways database^3^. Blue nodes are inputs, which are identified by searching for the keyword ‘Receptor’ in the UniProt entries of the genes associated with each node^27^. The red nodes are outputs, which are similarly identified by searching for the keyword ‘Transcription regulation’. Note that even with six canonical pathways the network is very interconnected and is difficult to globally comprehend. (**c**) Two human cell populations from different cell lines are exposed to the same input. However, while the majority of Population A proliferates in response to the input, the majority of Population B undergoes apoptosis.

To determine the variety of responses any given network can produce, we first generated the set of possible ‘expression vectors’ for that network. Each expression vector represents a unique pattern of presence or absence for each of the intermediate notes in the network; this is meant to represent all possible unique ‘cell types’ that could exist within this model, with each distinct cell type expressing a different subset of signalling proteins. For example a network with two intermediate nodes has a total of four different expression vectors: ‘00’, in which both are absent, ‘01’ and ‘10’, in which either is present, and ‘11’, in which both are present. In principle, each different expression vector could generate a network with a different response to the same input signals (for example, Fig. 1c). For each expression vector, we ran a Boolean network simulation with no inputs active, either input active, and both active, and measured the activity of the outputs at steady state. Note that, during these simulations, the nodes that are not expressed cannot become active, since the network cannot activate a protein that is not present within the cell. The activation state of all the expressed nodes, however, can evolve freely during the simulation according to a straightforward set of Boolean logic functions (see Methods and Supplementary Methods). This simulation produces a steady-state input/output (*I*/*O*) map for that expression vector, which is a string of 8 binary digits that represents the activity of both outputs in response to the four combinations of active inputs (that is, the input activities 00-01-10-11 might produce the *I*/*O* map 01-10-11-00). By applying these simulations to each unique expression vector, we can determine the total number of unique *I*/*O* maps the network is able to generate across all expression vectors.

**Figure 2:**
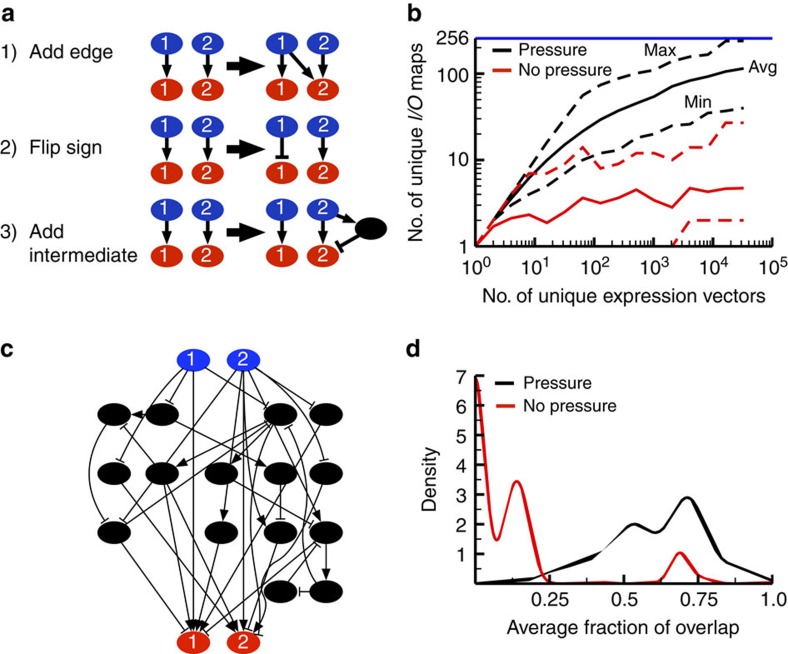
Evolvable Boolean signalling networks. (**a**) Diagram of the three possible modifications to the network. When an edge is added, the new edge connects one random node to another, and may be either activating or inhibitory. When an edge’s sign is flipped, a randomly chosen edge is switched from being activating to being inhibitory or vice-versa. When an intermediate is added, the intermediate connects one random node to another. The sign of the edges to and from the new node are chosen randomly. (**b**) The number (No.) of unique *I*/*O* maps as a function of the number of unique expression vectors. A network has 2^*N*^ unique expression vectors, where *N* is the number of intermediate nodes in the network. For networks with 2 inputs and 2 outputs, there are a maximum of 256 unique *I*/*O* maps possible. Two sets of networks were evolved: one set included an evolutionary pressure to obtain as many unique *I*/*O* maps as possible (black) while the other set was evolved randomly (red). (**c**) An example of an evolved signalling network with 15 intermediate nodes (32,768 unique expression vectors) and 33 edges connected the two inputs to the two outputs. This network generates 226 unique *I*/*O* maps, depending upon the expression of its intermediates. (**d**) The kernel density estimate of the average fraction of overlap (after compression, see Supplementary Methods) of 300 evolved networks with pressure to maximize the number of unique *I*/*O* maps (black) and without such pressure (red). Note that the average fractions of overlap were determined from fully evolved networks with 15 intermediate nodes.

We began this evolutionary algorithm with a TCS-like network where each pathway includes an input activating an intermediate node, which then activates an output. The TCS-like network has only four unique expression vectors, each of which produces a unique *I*/*O* map. However, as the model evolves and includes additional intermediate nodes, the network generates a larger number of *I*/*O* maps; networks with only 15 intermediate nodes are able to produce over 200 *I*/*O* maps (Fig. 2b). For example, the network diagrammed in Fig. 2c generates 226 distinct *I*/*O* maps, depending upon the expression of its intermediates.

Although the increase in *I*/*O* map diversity in these evolutionary simulations is striking, it is unclear if this is simply due to the increase in the number of nodes and edges in the network, or if it is a direct result of selection. To test this, we created a version of our evolutionary algorithm without the selection criteria; in this case, every randomly chosen alteration to the existing network was accepted regardless of its impact on the *I*/*O* maps. Using this altered algorithm, we produced a set of randomly evolved networks with up to 15 intermediate nodes. The lack of evolutionary pressure resulted in networks with relatively little *I*/*O* map diversity, generating an average of 10 unique *I*/*O* maps, an order of magnitude less than networks evolved with a pressure to diversify (Fig. 2b, red).

We found that networks evolved under the pressure to increase diversity bear a fairly striking resemblance to the types of networks found in metazoan cells (Fig. 2c versus Fig. 1b), exhibiting extensive crosstalk and complexity. This architecture allows for a very diverse array of responses depending on which nodes are present in the network. To quantify both network complexity and crosstalk, we employed a measure that we term the ‘average fraction of overlap.’ To do this, we first define all of the nodes downstream of any input *i* as *D*_*i*_, and the nodes upstream of any output *j* as *U*_*j*_. We then define the ‘pathset’ between *i* and *j* as the intersection of these two sets, *P*_*ij*_≡*D*_*i*_∩*U*_*j*_. This allows us to collect the set of all nodes that are in between input *i* and output *j* without restricting that set to some preconceived linear pathway (Figs 1b and 2c). The overlap between two distinct pathsets *P*_*ij*_ and *P*_*xy*_ is defined as fraction of nodes shared between them; the higher this number, the greater the tendency for input *i* to use the same set of nodes to reach output *j* that input *x* uses to reach output *y*. The average fraction of overlap is then calculated by averaging this quantity across all unique *ij*–*xy* pairs in the network. One concern with this method is that isolated cascades in a network may lead to spurious values of the average fraction overlap. To address this, we ran a compression algorithm on the network to collapse such cascades into a single node before we defined the pathsets and calculated the fractions of overlap (see Supplementary Methods for further details).

We found that the mean average fraction of overlap for networks evolved with a pressure to diversify was 0.61 while the mean average fraction of overlap for randomly evolved networks was significantly lower (0.12, *P*<2.2 × 10^−16^, Wilcoxon rank-sum test; Fig. 2d). This suggests that the pressure to increase *I*/*O* map diversity results in increase in crosstalk, though it is important to note that the correlation between *I*/*O* map diversity and average fraction overlap is not perfect (Supplementary Methods). Nevertheless, even in this simple model, networks with relatively high levels of crosstalk allow different cell types expressing different nodes in the network to readily exhibit widely different responses (or undertake different cell fate decisions) based on the same two input signals^26^.

### Expression patterns are selected for signalling diversity

In order to characterize the interconnectedness of the human signalling network and understand how this architecture and differential gene expression might affect responses to signals, we compiled a large Boolean network model by combining the contents of 29 signalling pathways from KEGG, resulting in a network with 735 nodes and 2,211 edges (see Supplementary Methods for details). Using UniProt we identified 76 input nodes (keyword: ‘Receptor’) and 67 output nodes (keyword: ‘Transcription regulation’)^27^. While KEGG provides information on whether any given interaction in the network is stimulatory or inhibitory, how those influences are integrated into the activity of any given protein in the network is unclear. In order to make construction and simulation of such a large network feasible, we developed a straightforward Boolean logic function to update the state of each node at each step, based on a set of fairly general regulatory principles in cell signalling (see Methods and Supplementary Methods for further details). While this is an abstraction of the true human signalling network, it allows us to focus on the impact of crosstalk and complexity on global input/output behaviour.

To highlight the relative complexity of this human signalling network, we drew a directed graph representing only a fraction of the total network (Fig. 1b). The resulting diagram is quite elaborate, with numerous edges connecting nodes from different sections of the system. Within the complete network, about 78% of the nodes are downstream of every single receptor, and every output is also downstream of every receptor, revealing a relatively high level of crosstalk between canonical pathways.

The complete network we compiled from KEGG, however, does not represent the signalling networks present within different cell types due to differences in expression of signalling proteins. In order to account for these differences, we obtained expression data for the network from the Human Protein Atlas^22^. This dataset includes relative expression levels (‘High’, ‘Medium’, ‘Low’ and ‘Not Detected’) based upon immunohistochemistry microarray assays for 344 of the 735 nodes in our network from 84 tissues. The remaining nodes either represent non-protein signalling elements, such as ions or small molecules, or were not included in the data set, usually due to experimental constraints (for example, the lack of a specific antibody for that protein). From the expression data we created 84 subnetworks where a node and its associated edges were removed if the node is ‘not detected’ in the respective tissue. The 391 nodes for which we have no direct expression data are always expressed.

**Figure 3:**
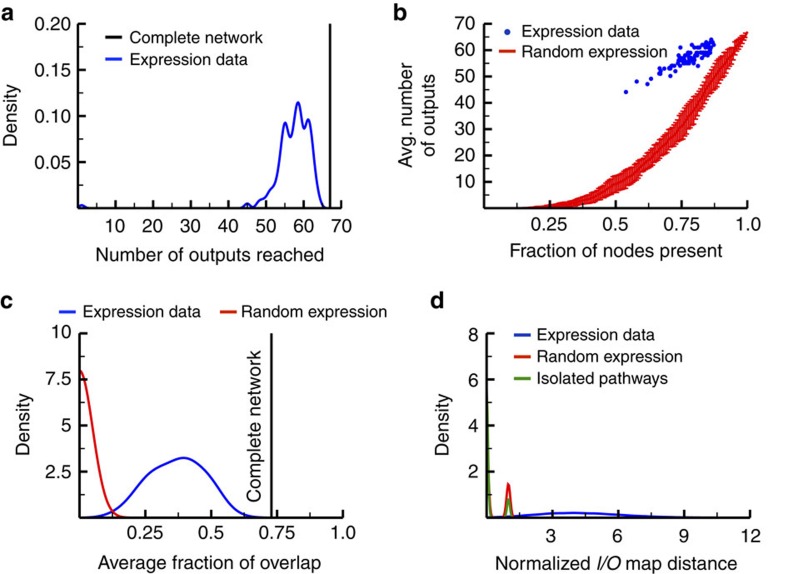
The structure and response diversity of the complete KEGG network. (**a**) The kernel density plot of the number of outputs that are downstream of each of the inputs in the complete network (black) and each of the expressed subnetworks (blue). All 67 outputs are downstream of each of the inputs in the complete network. However, most inputs have 50–64 of the outputs in their downstream connected component in the expressed subnetworks. (**b**) The average number of outputs that are downstream of each of the inputs in a subnetwork versus the fraction of nodes expressed in the subnetwork. The blue dots include the connectivity of the subnetworks constructed according to the expression data from the Human Protein Atlas^22^ while the red lines show the mean and standard deviation of 10 subnetworks constructed through the random expression of the indicated fraction of nodes. Note that the average number of outputs downstream of any input is much lower in the random subnetworks, indicating that these subnetworks demonstrate less interconnectivity than those based upon human expression data (minimum difference=9.398, *P*=1.546 × 10^−4^). (**c**) The kernel density plot of the average fraction of overlap for the expressed subnetworks (red) and the subnetworks from random expression of nodes (blue), after compression (after compression, see Supplementary Methods). The average fraction of overlap for the complete network is shown as a black vertical bar. (**d**) A kernel density plot of the normalized *I*/*O* map distances of the human subnetworks (blue), random subnetworks (red), and the TCS-like networks (green) comparing the output activities of the 84 subnetworks in response to a single active input.

These subnetworks all exhibit structures that are quite different from the complete network we originally compiled from KEGG: while each input can directly affect all 67 output nodes in the complete network, they can only reach about 50–64 outputs in the expressed subnetworks (Fig. 3a). Thus, while the complete network serves as a useful summary of the possible interactions among proteins in the human signalling network, differences in the presence or absence of various nodes clearly generates a diverse set of network topologies in various tissues.

The average number of outputs each input can reach in an expressed subnetwork strongly correlates with the fraction of nodes present in the subnetwork (Spearman’s *ρ*=0.88) (Fig. 3b, blue). To better understand subnetwork topology, we compared the human expressed subnetworks to a set of random subnetworks of varying sizes (Fig. 3b, red). For any given fraction of nodes expressed in these subnetworks, we chose the set of nodes to be expressed at random with uniform probability. As with the expressed subnetworks, any node not expressed in these random subnetworks was removed, along with its associated edges. As a result, these subnetworks are not randomizations of the network architecture, but rather just randomizations of which nodes in the graph are present or absent. For any given network size, it is clear that the expressed subnetworks found in human tissues maintain a significantly higher connectivity between inputs and outputs than random expression vectors (minimum difference=9.398, *P*=1.546 × 10^−4^). The expression of individual nodes in human tissues does not depend upon their indegree, outdegree, or their betweenness with regard to inputs to outputs, common properties that are used to quantify the ‘topological centrality’ of nodes in a network. This suggests that the non-random connectivity we observe depends on global properties of the set of nodes expressed in various human tissues (Supplementary Fig. 1).

We also found that the expressed subnetworks have less overlap between pathsets than the complete network. The complete network has an average fraction of overlap of about 0.73, indicating that most of the intermediate nodes exist on some path between any input to any output (Fig. 3c, black). The expressed subnetworks have a mean overlap of about 0.37, significantly lower than the overlap found in the complete network (Fig. 3b, blue *P*<2.2 × 10^−16^, Student’s *t*-test). To evaluate whether or not this difference was simply due to the fact that the expressed subnetworks are smaller than the full network, we performed a random control similar to that in Fig. 3b. In this case, however, rather than varying the number of expressed nodes from 0 to 1, we instead created a ‘random’ version of each human tissue, where the random version has precisely the same number of nodes expressed as the real tissue, but the specific nodes expressed are chosen at random. We created 10 independent sets of 84 tissues, and found that these random subnetworks exhibit significantly less overlap than the expressed subnetworks (Fig. 3c, red, mean≈0, *P*<2.2 × 10^−16^, Wilcoxon rank-sum test).

We then performed a set of Boolean network simulations on each subnetwork to evaluate whether their non-random architecture had an impact on their functional properties. For any given subnetwork, we independently activated each input and ran the model to steady state. To account for possible feedback loops, the ‘output’ state of any node in this case is taken as its average activity over the last 1,000 steps of the simulation. The *I*/*O* map for any given tissue in this case is the matrix of steady-state output values obtained across all possible inputs. We also defined an *I*/*O* map distance for a pair of tissues as the number of differences between the two *I*/*O* maps (see Supplementary Methods). We normalized the *I*/*O* map distance by the total number of input combinations possible, 76 choose *n*; by normalizing in such a way we get an idea of the average number of outputs whose activity are different between any two cell types. The higher the *I*/*O* map difference between two networks, the less similar their responses are across all possible inputs.

We determined the *I*/*O* map distances for all pairs of human subnetworks with one active input (Fig. 3d). As in Fig. 3c, we generated specific sets of 84 random subnetworks, with each random subnetwork in the set having the same number of nodes as one of the human networks. We generated ten sets of random networks, obtained the *I*/*O* map for each, and calculated the distances within each random set. The average normalized distance between the human subnetworks was around 4.3, yet the normalized distances for the random subnetworks was much closer to 0.31, indicating that the human subnetworks are not only more connected, but they also demonstrate a higher number of outputs with differing activity in response to the same input (Fig. 3d, *P*<10^−6^, two-tailed permutation test). To compare the human subnetworks to a TCS-like network consisting of truly isolated pathways, we created a network where inputs are connected to outputs through a linear succession of intermediate nodes. We did this by randomly adding each node from the KEGG network to one of the ‘pathways’ activated by one of the input nodes; after all intermediate nodes were added to the network, we terminated each pathway with an output node. This resulted in a network that included all the nodes in the KEGG network, but comprising 76 distinct pathways with absolutely no crosstalk. ‘Tissue-specific’ subnetworks of this TCS-like network were created using the expression data from the Human Protein Atlas. The normalized *I*/*O* map distances of the TCS-like network were even lower than those from the random subnetworks (average=0.13), indicating cells with isolated pathways show very little diversity (*P*<10^−6^ for the comparison with either the actual human networks or the random subnetworks, two-tailed permutation test).

Although the above analysis is informative, we only activated a single input at a time, and it is unclear that the same patterns of signalling diversity would be observed when multiple inputs are activated. To test this, we tested all combinations of 2 or 3 active inputs, resulting in 2,850 and 70,300 input combinations on the human subnetworks, random subnetworks, and TCS-like subnetworks. We found that the difference in normalized *I*/*O* distances between the three types of networks (that is, actual human tissues, random subnetworks and the TCS-like subnetworks) does not appear to depend on the number of inputs activated (see Supplementary Fig. 2, *P*<10^−6^, two-tailed permutation test, for the human networks having higher normalized distances than either control and the random subnetworks having higher distances than the TCS-like networks).

Our results indicate that the diversity of responses to input depends both on the complexity of the network and the targeted expression of particular sets of signalling proteins within various cell types. Interestingly, signalling diversity does not seem to be dependent only on the number of nodes in the network, since then random subnetworks have the same size, but the human subnetworks possess a higher average *I*/*O* map distance. Instead, diversity is generated by the entire context of nodes expressed in each tissue.

### Differential effects of inhibitors across cell types

An interesting observation from our evolved Boolean networks (Fig. 2) is that, despite the tens of thousands of possible expression vectors, these subnetworks only produce an average of around 100 unique *I*/*O* maps (Fig. 2b). This means that many subnetworks must produce the same *I*/*O* map, and in most cases adding or removing any given node has no effect on the ‘function’ of a particular expressed network. The network topologies in this case were evolved purely based on their ability to generate multiple *I*/*O* maps, so this relative ‘robustness’ emerges in the absence of any explicit evolutionary pressure. Previous studies have found similar behaviors in complex biological systems: for instance, many different ‘genotypes’ (for example, RNA or amino acid sequences) may have the same ‘phenotype’ (for example, folded, three-dimensional structure). Such systems often evolve structures that are robust to mutations, even if considerations of robustness or evolvability are not used as an explicit selection criterion^28^,^29^,^30^,^31^,^32^,^33^,^34^.

**Figure 4:**
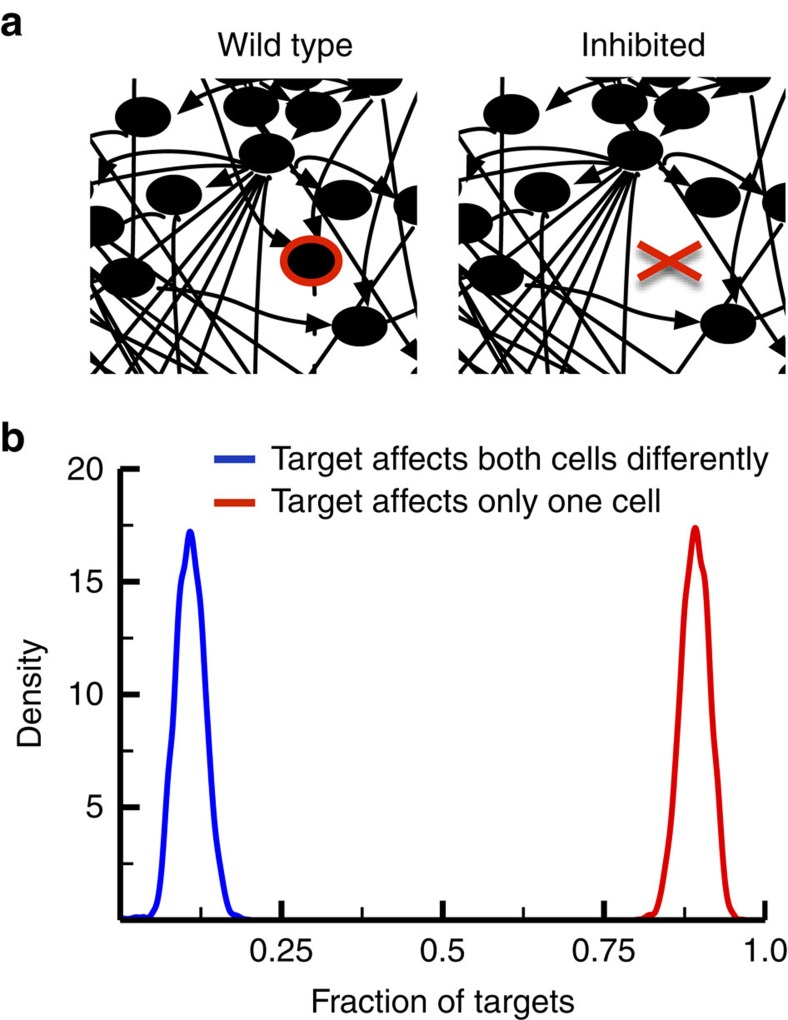
The effects of inhibitors on different cells. (**a**) Each of the nodes in complete the network was targeted for inhibition. When a node is targeted for inhibition (that is, the red highlighted node in the wild-type panel), the node and its associated edges (including both incoming and outgoing edges) are removed from the expressed subnetwork. (**b**) A kernel density plot of the fraction of nodes whose inhibition changes the response to input in either of pair of subnetworks. Only about 12% of the targetable nodes affect both cells, and inhibiting each of these nodes affects both of the cells differently. The remaining targetable nodes affect only one cell, either because the nodes are not expressed in the other cell or because the other cell includes connections within the network that can compensate for the actions of the inhibitor.

In the case of the human expressed subnetworks, this robustness would imply that inhibiting a protein (for example, using a small molecule or drug, or knocking it down through RNAi) might have different effects in different tissues. We explored this possibility by sequentially removing each node from all of our 84 tissue-specific network; when any given node is removed, this represents its complete inhibition in that tissue (Fig. 4a). We then obtained the *I*/*O* maps for every inhibited system, which we compared to the wild-type *I*/*O* map for the respective expressed subnetwork. From this comparison we obtained an inhibitor map, where a ‘1’ represents an increase in the average activity for an output, a ‘−1’ represents a decrease in average activity, and a ‘0’ signifies no change in average activity. We found that every single node has an effect on the *I*/*O* map of at least one tissue when inhibited, so none of the nodes are completely dispensable. Also, every node had at least one tissue where inhibition of the node had no effect. On average, inhibiting any node in the network alters the output response in about 17 of the 84 human expressed subnetworks (Supplementary Fig. 3).

The inhibitor maps for each targeted node across all pairs of expressed subnetworks were then analyzed to characterize the effects of inhibiting the respective node on each tissue. Of the subset of nodes whose inhibition had an effect on the *I*/*O* map of either subnetwork across all pairs, the inhibition of about 88% of the targets affected only one of the subnetworks (Fig. 4b, red). This could be because the node is not expressed in the other subnetwork, so the inhibitor had nothing to target, or because the other subnetwork possesses redundancy in its structure so that removal of that node has no effect. Inhibiting the remaining 12% of the targets affected both of the subnetworks, yet resulted in a different effect in each (Fig. 4b, blue). We found no cases where the inhibition of a single node had the same impact in two subnetworks.

These results may help explain previous experimental findings for drugs and inhibitors targeted against signalling proteins. For instance, Fallahi-Sichani, *et al*.^35^, recently tested the efficacy of various anticancer drugs against a panel of breast cell lines; drugs targeting signalling proteins generally exhibited a higher variation in efficacy against different cell lines compared to those targeting more ubiquitous targets such as the proteasome or DNA. Although all of the cell lines in this case derive from the same tissue, variations in expression levels of signalling proteins may well influence how inhibition of those proteins effects cellular decisions such as division or apoptosis. The massive amount of crosstalk in the human signalling network may also underlie the ability of certain cancer cells to compensate for drug effects^36^. Nutlin-3, for example, was designed to be a competitive inhibitor of the p53-MDM2 interaction in order to induce apoptosis or senescence^23^. However, nutlin-3 only results in reversible cell cycle arrest in most cell lines expressing wild-type p53; the induction of apoptosis or senescence is dependent upon the overexpression of MDM2 (refs 23, 37, 38, 39). Tissue selectivity may have consequences for the use of inhibitors and gene silencing in future signalling studies: these perturbations could have unexpectedly different effects in various cell lines. Care must be taken to consider the tissue-specific context of the signalling network to inform the interpretation of inhibitor or silencing experiments.

## Discussion

Although individual cells that belong to the same cell type can exhibit significant variation, the average protein levels within the signalling network of those cells ultimately leads to a particular average response within a population^40^,^41^,^42^. In this work, we demonstrated that these different expression patterns likely have large impacts on the function of the signalling networks within these cells. In particular, we found that subnetworks specific to various human tissues are highly complex, yet the global topology of the network exhibits significant differences across different tissues. Moreover, the expression of particular nodes does not seem to be random, nor is it dependent upon classic individual properties of the node such as degree or betweenness. The set of expressed nodes have instead likely evolved to maintain crosstalk between inputs and outputs and to produce a diversity of responses to stimuli (Fig. 3). While our ‘average fraction overlap’ measure provides some insight into the relationship between network topology and *I*/*O* map diversity (Figs 2 and 3), considerable future work will be necessary to understand the specific structural features of the network, or the expressed subnetworks, that underlie functional diversity.

A consequence of the variable architecture of the human signalling network across tissues is that perturbations to the network, such as small molecule inhibition, may affect those tissues or cell types in different ways. The local context of a targeted protein may be very different in different cells based on the expression state of its signalling neighbours. We find that targeting any node for inhibition in the network can potentially change the response of a cell to stimuli in some, but not all, tissues. However, the phenotypic consequences of the inhibitor vary between the different cell types it affects. In our models, we found no examples of an inhibitor that would have the same effect in two or more tissues in our subnetwork, revealing the importance of tissue-specific context in considering drug targets.

These findings may also provide an explanation for the variety of mutations that are associated with cancers and other diseases. Protein affecting mutations (PAMs) can perturb signalling networks by changing the activity or expression of a particular protein. It has been found that a PAM may not have the same phenotypic effect in different tissues, similar to how inhibitors have different effects in our tissue-specific subnetworks. For instance, mutations affecting p53 and PIK3CA, both high-confidence drivers (HCDs) of carcinogenesis, have been found in just over 10% of cancer samples. In fact, mutations in several HCDs have been discovered in only a single tumor type^43^. Our results suggest that mutations to a protein in some cell types and tissues might have no effect on the signalling properties of those cells, while in other cell types that same mutation might lead to aberrant (that is, carcinogenic or pathogenic) signalling changes.

Overall, our work indicates that the highly interconnected architecture of metazoan signalling networks has likely evolved based on the need for multiple cell types and tissues to respond differently to the same environmental conditions. Understanding the consequences of this constraint for network topology, target selection and pathogenic mutations represents a major challenge for systems biology and pharmacology.

## Methods

### Evolvable Boolean networks

A given Boolean network was randomly altered through one of three possible modifications: (1) adding an edge, (2) flipping an edge from activating to inhibiting or vice versa, or (3) adding an intermediate nodes to randomly connect two existing nodes. Following a modification, the network was simulated with one, both, or neither of the inputs active using a custom synchronous Boolean simulator for 100 steps. We then combined the final activity of the outputs to form an 8 digit binary string (that is, ‘00101101’), which we term the ‘*I*/*O* map’. If the network has one or more intermediates, we then run the set of simulations to obtain an *I*/*O* map for every possible expression vector; for instance, a network with two intermediate nodes has four expression vectors: both expressed, one expressed, the other expressed, and neither expressed. Acceptance of a modification depends upon the number of unique *I*/*O* maps across all expression vectors (Supplementary Methods).

### The KEGG network

The complete KEGG signalling network was constructed from KGML files from 29 canonical pathways^3^. These pathways were initially expanded so that each gene or compound in an entry existed as its own node. This also expanded the number of edges from each pathway so that each node from an entry had its own set of edges. For example, if entry 2, which includes two genes, activates entry 3, which includes four genes, then this edge was expanded to include 8 different gene pairs. Once the full network was built from the different pathways, nodes that are involved in the same set of edges were collapsed into a single node.

### Boolean simulations

While the KEGG database provides information on whether an interaction between two proteins is activating or inhibiting, it does not provide Boolean functions describing how these influences are integrated into the ultimate activation state of any given node in the network. Considering the size of the network, and the potential complexity of the evolvable Boolean networks, we generated a basic ansatz for the Boolean update functions used in our simulations. We made the simplifying assumption that each activator for a node acted in the same manner (for example, multiple kinases phosphorylating the same set of sites). As such, having any number of functionally active activators would ‘turn on’ that node. The only exception to this would be nodes that have no activators that act upon them; these are considered to be self-activating. On the other hand, we assumed that repressors resulted in the wholesale deactivation of the node no matter the state of the node’s activators. This is due to the fact that many inhibitors lead to irreversible degradation of a protein; An example would be the serine/threonine phosphorylation of the insulin receptor substrate-1 (IRS-1) by S6K1, among other serine kinases, which leads to ubiquitylation and degradation of IRS-1 (refs 44, 45). As such, if any of the activators of a node at step *i* of the simulation, or if the node has no activators, and none of the repressors are active, then the node will be active at step *i+*1. If none of the activators, if there are any for the node, are active at step *i*, or if any of the repressors are active at step *i*, then the node will be inactive at step *i+*1. A more formal description of this logic function can be found in the Supplementary Methods.

### Tissue-specific subnetwork simulations

We obtained the expression of each of the genes in the network in 84 tissue types from the Human Protein Atlas^22^. The expression of any particular node is dependent upon the expression of each of its associated genes: if any gene included in the node is expressed at any level within a tissue, then the node is expressed in that tissue. The expression state for each of the genes in the network for any of the 84 tissue types represents a single expression vector. When an expression vector is applied to the complete KEGG network, nodes that are not expressed in the tissue are removed from the network, resulting in a tissue-specific subnetwork.

To obtain an *I*/*O* map distance between two subnetworks for a particular set of active input vectors, we ran each subnetwork for 10,000 steps in a custom synchronous Boolean simulator. The *I*/*O* map in this case is the set of the average activity of each output in the last 1,000 steps of the simulation for each active input vector. The result is a matrix where each row contains 67 values from 0 to 1 for the average activity of each output in response to the activation of a set of inputs. The individual elements from the matrices of the two subnetworks were then compared to obtain the distance: if the elements did not match, the distance was increased by 1 (see Supplementary Methods for more information).

### Data availability

The data that support the findings of this study are available from the corresponding author upon reasonable request.

## Additional information

**How to cite this article:** Rowland, M. A. *et al*. Crosstalk and the evolvability of intracellular communication. *Nat. Commun.* **8**, 16009 doi: 10.1038/ncomms16009 (2017).

**Publisher’s note:** Springer Nature remains neutral with regard to jurisdictional claims in published maps and institutional affiliations.

## Supplementary Material

Supplementary Information
