# Supplementary material for: Crosstalk and the evolvability of intracellular communication
Source: Nat Commun. 2017 Jul 10;8:16009. doi: 10.1038/ncomms16009 (PMC5508131; doi:10.1038/ncomms16009)
Supplement: Supplementary Information [file ncomms16009-s1.pdf]

Type of file: PDF
Size of file: 0 KB
Title of file for HTML: Supplementary Information
Description: Supplementary figures, supplementary tables, supplementary methods and supplementary references.

Supplementary Figures

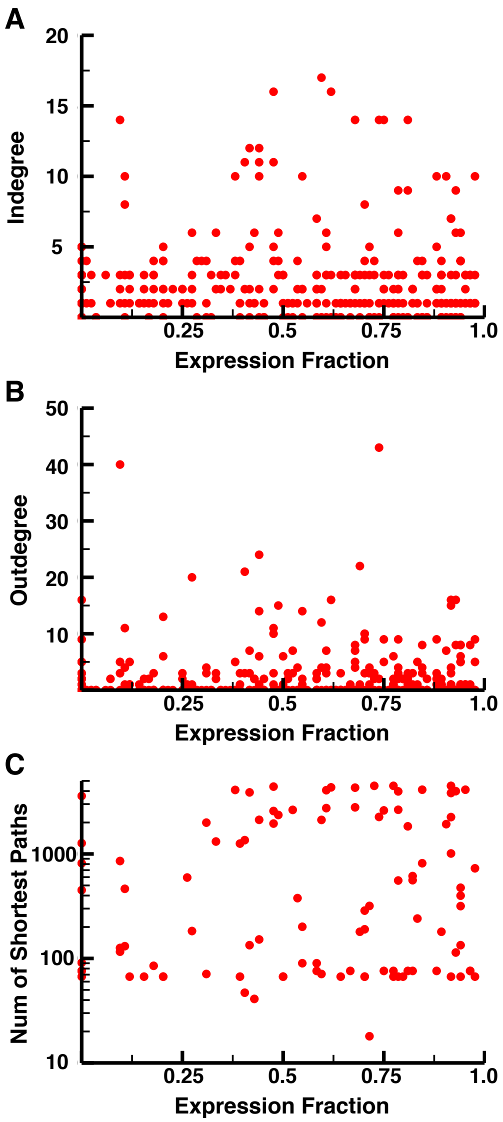

Supplementary Figure 1: Node properties versus the expression fraction of a node. (A) The indegree of a node versus its expression fraction. The expression fraction of a node is calculated as the ratio of the number of tissues in which the node is expressed to the total number of tissues. There is no correlation between the indegree and the expression fraction (Spearman's $\rho = 0.03824749$, p = 0.4795). (B) The outdegree of a node versus its expression fraction. There is a weak correlation between the outdegree and the expression fraction (Spearman's $\rho = 0.15365$, p = 4.285 $\times 10^{-3}$), however the data cannot provide a statistically significant linear regression (p = 0.1548, adjusted R-squared = 0.003004). (C) The number of shortest paths between an input and an output that includes a node versus its expression fraction. There is a weak correlation between the number of shortest paths and the expression fraction (Spearman's $\rho = 0.1506653$, p = 5.106 $\times 10^{-3}$), however the data cannot provide a statistically significant linear regression (p = 0.5059, adjusted R-squared = 0.005518).

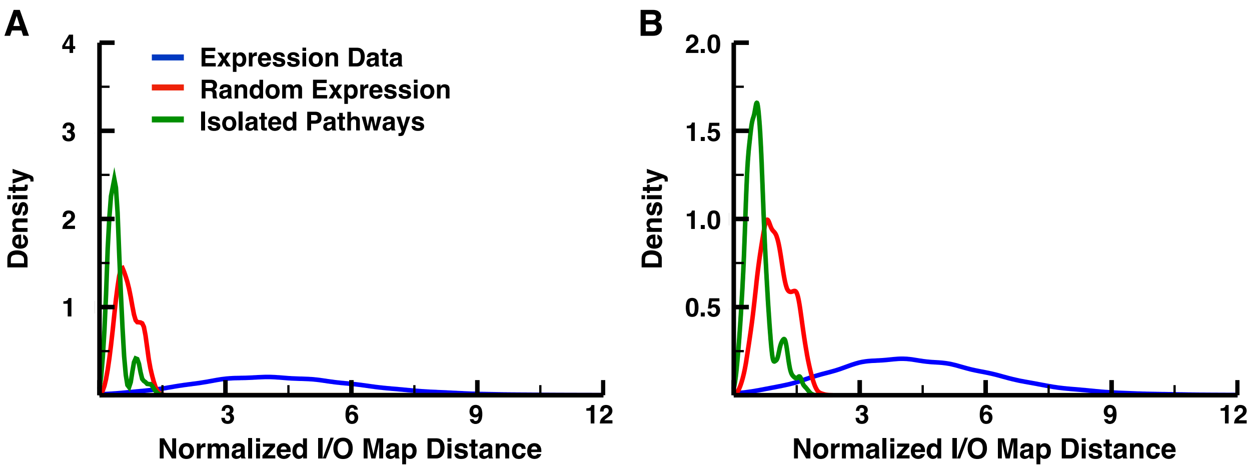

Supplementary Figure 2: Kernel density plots comparing the normalized I/O map distances of human subnetworks (blue), random subnetworks (red), and TCS-like networks (green) with two (*A*) or three (*B*) active inputs.

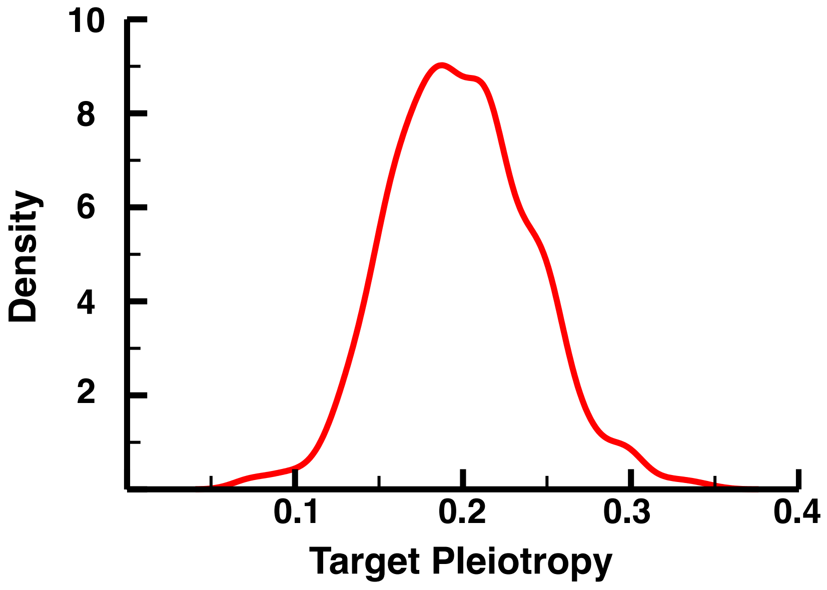

Supplementary Figure 3: Kernel density plot of target pleiotropy across the 84 expressed subnetworks. The target pleiotropy is the fraction of the 84 tissues in which inhibiting the node results in a change in output activity. Note that there are no nodes whose inhibition has an effect in all 84 tissues (Target pleiotropy = 1). Additionally, every node can be targeted in order to have an effect in at least one tissue. On average, an inhibitor will alter the output activity, and potentially change the phenotypic response of the cell, in about 17 of the 84 tissues (~20%).

Supplementary Methods

Evolvable Boolean Networks

The evolvable Boolean networks start out with the same topology: two inputs (blue) that each activate their associated output (red) (Fig. 4). These networks are randomly altered through one of three possible modifications: (1) adding an edge, (2) flipping an edge from activating to inhibiting or vice-versa, or (3) adding an intermediate node to connect two random nodes (See Fig. 2*A* of the main text).

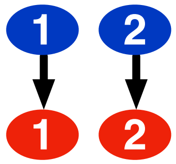

Supplementary Figure 4: Diagram of the initial TCS-like Boolean network

Following a modification, we run a synchronous Boolean simulation of the network for 100 steps with one, both or neither of the inputs active throughout the simulation. We then obtain the final activity of both outputs from each of the four simulations (Table 1). These output activities are combined to form an 8 digit binary string, the 'I/O map' (i.e., the I/O map from Table 1 would be '01110111').

		Input 1 and Input 2 Activity			
		I_1I_2	I_1I_2	I_1I_2	I_1I_2
		00	10	01	11
Output Activity	O_1	0	1	0	1
	O_2	1	1	1	1

Supplementary Table 1: The final activity of outputs O_1 and O_2 for a modified network after a 100 step synchronous Boolean simulation. Each of the four rightmost columns represent the four different combinations of input activity, with neither input, input I_1, input I_2, or both inputs active throughout the simulation, respectively. From these results we would obtain the I/O map '01110111', which is the output O_1 and O_2 activities for each of the input combinations.

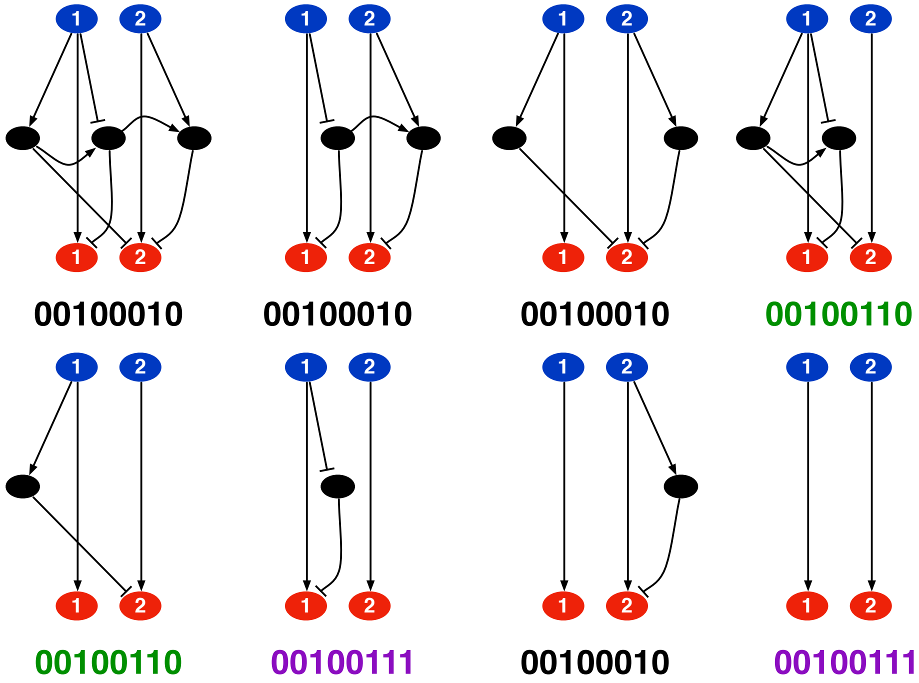

Supplementary Figure 5: Diagrams of each of 8 possible expressed subnetworks for a hypothetical evolved network with three intermediate nodes. Each subnetwork exhibits a unique expression vector, which is to say that they have a unique combination of the three intermediate nodes. Beneath each subnetwork is the associated I/O Map, with different colors representing distinct maps. This example network thus presents 3 unique I/O Maps.

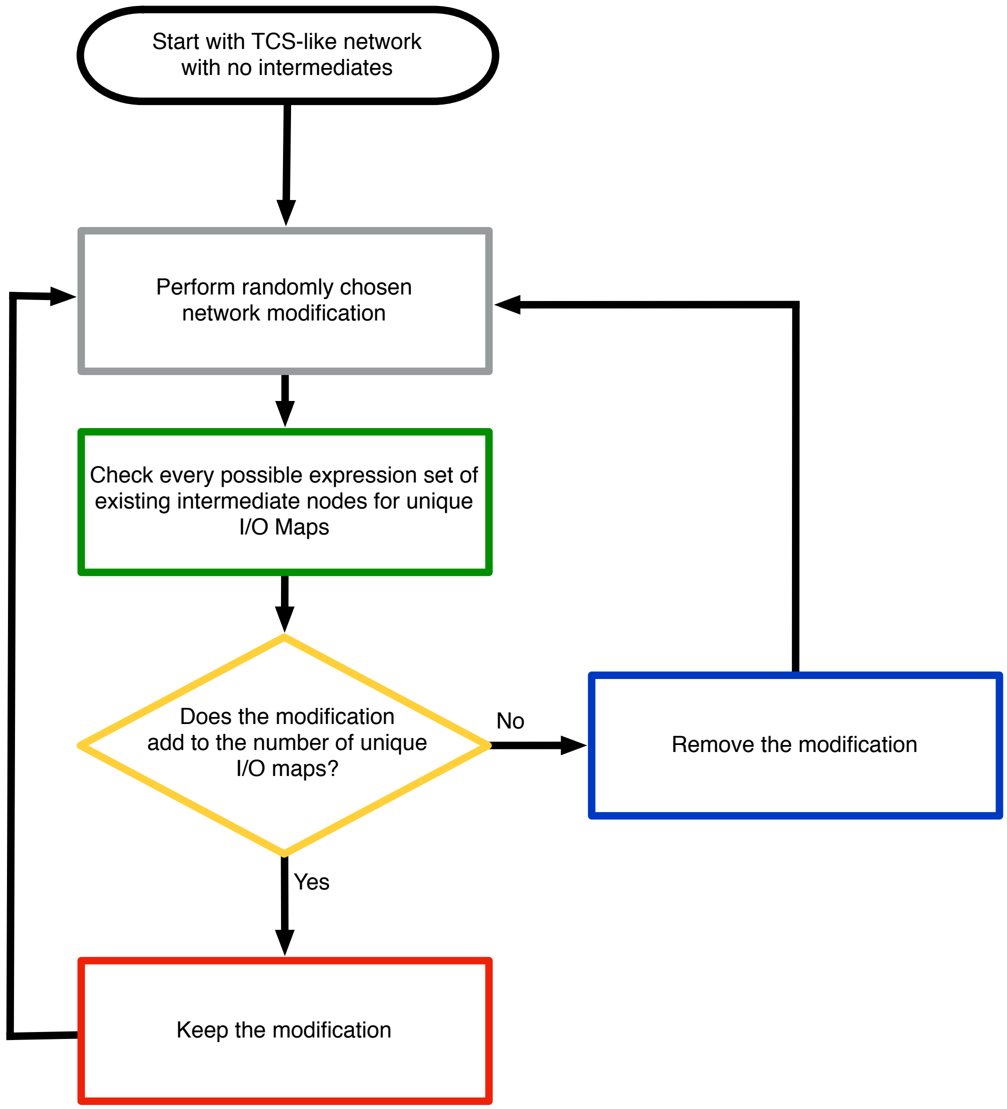

Supplementary Figure 6: The flow chart for the evolution of the Boolean signaling networks. After a modification is made to the existing network, the intermediate nodes are variably expressed in every possible combination. We then count the number of unique I/O maps and keep the modification if it increases the number of unique maps. Otherwise the modification is removed and the process is started again.

In order to explore the full potential of these systems, if a network has one or more intermediates, then we obtain the I/O map for every combination of expressed intermediate nodes, which we term an 'expression vector'. For example, a network with three intermediate nodes will have $2^3 = 8$ possible expression vectors, depending upon the presence or absence of each of the intermediates (see Fig. 5). Due to the differences in network architecture, the network resulting from an expression vector has the potential to produce a unique I/O map. For a system with two inputs and two outputs, there is a maximum of 256 unique I/O maps possible. After each modification, we obtain the I/O map for each expression vector and count the number of unique I/O maps produced by the network. The number of unique maps is used to determine whether

the modification is accepted: if the number of unique maps is increased by the modification, it is kept. New modifications are attempted until the network has 16 intermediate nodes. See Fig. 6 for the flow chart of the evolutionary algorithm.

Boolean Simulations

We ran synchronous Boolean simulations of the networks presented in this work to determine the functional impact of the complexity of the network on the activity of the output nodes. Input nodes are assumed to be either constantly active or constantly inactive throughout a simulation. The activity of the intermediate nodes are updated each step according to:

$$
\begin{aligned}
Int_i = ((&Activator_a \lor Activator_b \lor ... \lor Activator_N) \\
&\land \neg(Repressor_a \lor Repressor_b \lor ... \lor Repressor_M)) \\
&\land Expression_i
\end{aligned}
$$

Where Int_i is an intermediate node with N upstream nodes that stimulate its activity and M upstream nodes that repress its activity. $Expression_i$ is the expression state of node i; if the node is expressed, that state is 1, otherwise it is 0. Biologically, this means that a node will be active at step $k+1$ of the simulation if it is expressed in the cell and if *at least* one of its activators is active, and *none* of its repressors are active, at step k. Otherwise the node will be inactive. The one exception to this is the scenario in which the intermediate node is only acted upon by repressors, in which case the logical expression becomes:

$$Int_j = (\neg(Repressor_a \lor Repressor_b \lor ... \lor Repressor_M) \land Expression_j$$

The activity of the outputs is determined in a similar fashion with the exception being that they are assumed to be constitutively expressed.

For each simulation, the activity of the intermediate nodes and outputs nodes are initialized to 0, and the inputs are initialized to 0 or 1 depending on the inputs being activated in that particular simulation. Simulations are run for 10,000 synchronous updates, meaning that the state of every node for step $i+1$ is updated based upon the state of the network for step i.

Average Fraction of Overlap

To address the problem of quantifying the "degree of crosstalk" within a network, we propose a more principled definition. To do this, we first define something we call a "pathset", which is the set of all nodes that are "in between" any given receptor (i.e. input) and transcription factor (i.e. output). More formally, we first defined D_i as the set of intermediate nodes in the downstream connected component for each input i. Note that here we use "downstream connected component" to indicate that, when we define this component, we start at input node i and find all nodes that can be reached only when following the directed edges in the graph in the forward direction. Similarly, we define U_j as the set of intermediate nodes in the upstream connected component for each output j. The pathset P_{ij} defined by any pair of inputs and outputs is then

defined as the intersection of these two sets, $P_{ij} \equiv D_i \cap U_j$. Any intermediate node in P_{ij} is on at least one path linking input i to output j; this allows us to define the nodes in each input and output without restricting this set to be some linear pathway.

To quantify the level of crosstalk in a given network, we focused on the overlap between a pair of distinct pathsets P_{ij} and P_{xy}, where i could be the same as x or j could be the same as y, but not both simultaneously. Specifically, we define the overlap between two such pathsets as the size of the intersection of P_{ij} and P_{xy} divided by the union of P_{ij} and P_{xy}; this is just the Jaccard index between the two sets. The average of this overlap indicates the extent to which the interactions that determine input/output relations are largely shared, or largely distinct, between pairs of inputs and outputs in the network.

One concern with the average fraction of overlap is that long cascades of proteins shared between multiple pathsets may cause the metric to overestimate the crosstalk within the network. Take, for example, the networks:

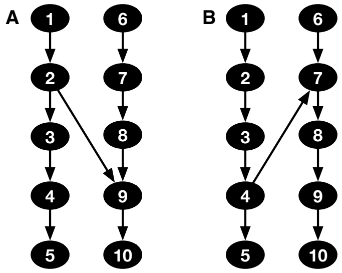

Both Networks A and B represent two cascades with a single interaction between them. The average fraction of overlap for these two networks are 0.08 and 0.17; these networks have different values quantifying "crosstalk" even though the amount of crosstalk seems very similar. This suggests that the average fraction of overlap might not be an ideal method for quantifying crosstalk. It is important to not that, while Networks A and B look similar at first glance, they are not functionally equivalent. These networks do not produce the same number of unique I/O maps, with Network A demonstrating 6 unique maps while Network B demonstrates only 4, dependent on which of the intermediate nodes (2-4, 7-9) are expressed. This highlights the fact that intuitive notions of crosstalk can have difficulty capturing relevant functional details, which is perhaps one of the reasons that developing a universally accepted quantification of crosstalk in biological networks has so far proved elusive.

For a second example, take the networks:

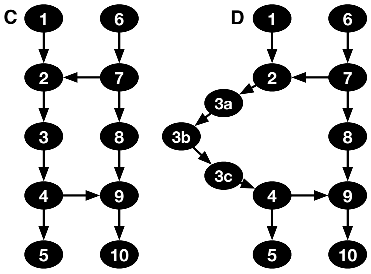

Networks C and D represent either two cascades of the same (C) or different lengths (D) with similar crosstalk connecting the cascades. The average fractions of overlap for these networks are 0.656 and 0.751. Unlike Networks A and B, Networks C and D are functionally equivalent from the standpoint of each demonstrating the same set of unique I/O maps.

The above examples suggest that applying the average fraction overlap directly to a network topology might result in spurious differences in the measured amount of crosstalk. This problem largely stems from isolated successions of interacting nodes in a cascade (e.g.nodes 3a, 3b and 3c in Network D). To address this issue, we developed an algorithm to "compress" the network - that is, to shorten instances of isolated cascades of interactions to a single node so that they do not have a strong impact on our calculation of the overlap between pathsets.

The compression algorithm first identifies the set of compressible nodes. These nodes have an in degree of 1, out degree of 1, are not directly acted upon by an input, and are not directly acted upon by an output. The last two criteria were included to make sure that there remains at least one node that would be considered an intermediate node in between any given input and any output. Since intermediate nodes are the only nodes that we allow to be differentially expressed when calculating I/O maps, this criterion minimizes the impact of the compression step on the number of unique I/O maps a particular topology can generate. Each compressible node is then replaced in the network by an interaction between the node directly upstream of the compressible node and the node directly downstream. In other words, we "cut" nodes that are found along pathways out of the network. The resultant network is then used in the calculation of the average fraction of overlap.

Nodes 3 and 8 (green) in Networks A and B are compressible - their removal leaves us with the networks:

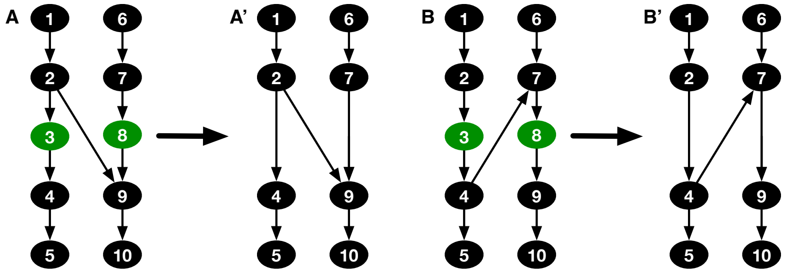

After the compression, the average fractions of overlap for Networks A' and B' are 0.1 and 0.17, which is closer than those for the original Networks A and B. However, we note that the average of fraction overlap, even with compression of the networks, is not a perfect metric of crosstalk. We should note that, in this case, Networks B and B' have higher average fractions of overlap despite having a smaller number of unique I/O maps. While we find a general increase in average fraction overlap with increasing I/O map diversity (e.g.Fig.2D in the main text), this correlation is clearly not perfect, even after compression. Considerable future work will be clearly be necessary to fully understand the relationship between network topology and function.

Networks C and D also have nodes that are compressible (green):

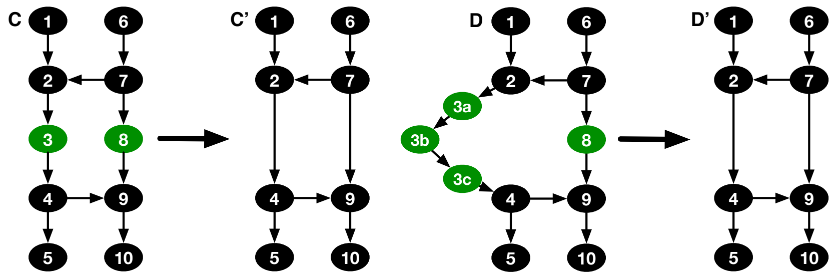

We can clearly see that the average fractions of overlap for the compressed networks C' and D' are the same as they are the same network (0.667). The compression algorithm deals with cases in which long, isolated cascades might result in spurious fraction overlap differences (as with Networks C and D) while still preserving sufficient topological information to capture functional differences (Networks A and B). As such, we applied this compression to all of the networks we consider before calculating the fraction overlap.

The Complete KEGG Signaling Network

We compiled the complete KEGG signaling network from the contents of the KGML files of 29 canonical pathways found in the KEGG Pathways database [1] (Table 2). These pathways included node entries such as:

```
<entry id="6" name="hsa:4790 hsa:5970" type="gene"
       link="http://www.kegg.jp/dbget-bin/www_bget?hsa:4790+hsa:5970">
       <graphics name="NFKB1, EBP-1, KBF1, NF-kB1, NF-kappa-B, NF-kappaB,
             NFKB-p105, NFKB-p50, NFkappaB, p105, p50..."
             fgcolor="#000000" bgcolor="#BFFFBF"
             type="rectangle" x="984" y="311" width="46" height="17"/>
  </entry>
```

where the 'name' attribute includes the KEGG entry for different isoforms of the same protein, in this case for NFκB1 and NFκB3. Each of these node entries are separated so that each KEGG entry becomes its own node, with each of these daughter nodes participating in the same incoming and outgoing edges. Once the nodes and edges from each of the canonical pathways were added, we collapsed the network by combining similar nodes. If two nodes had the same incoming and outgoing edges, these nodes were then combined into a single node. This process was done iteratively until there were no more nodes that could be combined.

For each of the nodes, we used the KEGG entries from each node to obtain the associated UniProt and ENSEMBL accession numbers. These were used to look up the expression data for the proteins included within each node from the Human Protein Atlas dataset [2]. The expression of any node in each of the tissues is dependent upon the expression of the genes associated the the node. If all of the associated genes are not expressed in a particular tissue, then the node is counted as not being expressed. However, if any of the genes are expressed, then the node is counted as being expressed in the tissue. This resulted in 84 expression vectors for the complete KEGG signaling network, generating 84 'expressed subnetworks', which represent the signaling

Pathway	URL
Rap1	http://www.kegg.jp/kegg-bin/show_pathway?map=hsa04015
RAS	http://www.kegg.jp/kegg-bin/show_pathway?map=hsa04014
MAPK	http://www.kegg.jp/kegg-bin/show_pathway?map=hsa04010
ErbB	http://www.kegg.jp/kegg-bin/show_pathway?map=hsa04012
WNT	http://www.kegg.jp/kegg-bin/show_pathway?map=hsa04310
TGF-β	http://www.kegg.jp/kegg-bin/show_pathway?map=hsa04350
VEGF	http://www.kegg.jp/kegg-bin/show_pathway?map=hsa04370
TNF	http://www.kegg.jp/kegg-bin/show_pathway?map=hsa04668
HIF-1	http://www.kegg.jp/kegg-bin/show_pathway?map=hsa04066
NFκB	http://www.kegg.jp/kegg-bin/show_pathway?map=hsa04064
Jak-STAT	http://www.kegg.jp/kegg-bin/show_pathway?map=hsa04630
FoxO	http://www.kegg.jp/kegg-bin/show_pathway?map=hsa04068
Calcium	http://www.kegg.jp/kegg-bin/show_pathway?map=hsa04020
PI3K - Akt	http://www.kegg.jp/kegg-bin/show_pathway?map=hsa04151
mTOR	http://www.kegg.jp/kegg-bin/show_pathway?map=hsa04150
Toll-like Receptor	http://www.kegg.jp/kegg-bin/show_pathway?map=hsa04620
NOD-like Receptor	http://www.kegg.jp/kegg-bin/show_pathway?map=hsa04621
T-cell receptor	http://www.kegg.jp/kegg-bin/show_pathway?map=hsa04660
B-cell Receptor	http://www.kegg.jp/kegg-bin/show_pathway?map=hsa04662
Fcϵ RI	http://www.kegg.jp/kegg-bin/show_pathway?map=hsa04664
Fcγ R	http://www.kegg.jp/kegg-bin/show_pathway?map=hsa04666
Chemokine	http://www.kegg.jp/kegg-bin/show_pathway?map=hsa04062
Insulin	http://www.kegg.jp/kegg-bin/show_pathway?map=hsa04910
Adipocytokine	http://www.kegg.jp/kegg-bin/show_pathway?map=hsa04920
GnRH	http://www.kegg.jp/kegg-bin/show_pathway?map=hsa04912
Prolactin	http://www.kegg.jp/kegg-bin/show_pathway?map=hsa04917
Estrogen	http://www.kegg.jp/kegg-bin/show_pathway?map=hsa04915
Oxytocin	http://www.kegg.jp/kegg-bin/show_pathway?map=hsa04921
Neurotrophin	http://www.kegg.jp/kegg-bin/show_pathway?map=hsa04722

Supplementary Table 2: The list of 29 canonical pathways in the KEGG Pathways database that were compiled to create the complete signaling network

network as it is expressed in each of the tissues. We then used the associated UniProt accession numbers to potentially annotate a node as an input (One UniProt entry includes the keyword 'Receptor') or as an output (One UniProt entry includes the keyword 'Transcription regulation') [3].

One of the metrics we use to compare subnetworks is the I/O map distance. To obtain the map distance we run each network in a synchronous Boolean simulation for 10000 steps with a set of N inputs active throughout the simulation. This is then done either for each of the inputs being activated individually (Fig. $3C$ of the main text) or for 50 combinations of N inputs (Fig. $3D$ of the main text). The activity of each output is averaged over the final 1000 steps to account for any oscillations in activity. This results in a matrix where each row is the average activity for all 67 outputs in response to a combination of active inputs. These respective elements in each of the matrices generated by the two subnetworks were then compared: the I/O map distance is the number of elements in the matrices that do not match.

Supplementary References

1. Kanehisa, M., Goto, S., Kawashima, S., Okuno, Y. & Hattori, M. The kegg resource for deciphering the genome. *Nucleic acids research* **32**, D277–80 (2004).

2. Uhlen, M. *et al.* Towards a knowledge-based human protein atlas. *Nature biotechnology* **28**, 1248–50 (2010).

3. Consortium, T. U. Update on activities at the universal protein resource (uniprot) in 2013. *Nucleic Acids Research* **41**, D43–7 (2013).
